# The Complete Genome Sequence of *Methanobrevibacter sp.* AbM4

**DOI:** 10.4056/sigs.3977691

**Published:** 2013-05-25

**Authors:** S. C. Leahy, W. J. Kelly, D. Li, Y. Li, E. Altermann, S. C. Lambie, F. Cox, G. T. Attwood

**Affiliations:** 1 New Zealand Agricultural Greenhouse Gas Research Centre; 2 Rumen Microbiology, Animal Nutrition and Health, AgResearch Limited, Grasslands Research Centre, New Zealand.

**Keywords:** Methanogen, methane, ruminant, *Methanobrevibacter*

## Abstract

*Methanobrevibacter sp.* AbM4 was originally isolated from the abomasal contents of a sheep and was chosen as a representative of the *Methanobrevibacter wolinii* clade for genome sequencing. The AbM4 genome is smaller than that of the rumen methanogen *M. ruminantium* M1 (2.0 Mb versus 2.93 Mb), encodes fewer open reading frames (ORFs) (1,671 versus 2,217) and has a lower G+C percentage (29% versus 33%). Overall, the composition of the AbM4 genome is very similar to that of M1 suggesting that the methanogenesis pathway and central metabolism of these strains are highly similar, and both organisms are likely to be amenable to inhibition by small molecule inhibitors and vaccine-based methane mitigation technologies targeting these conserved features. The main differences compared to M1 are that AbM4 has a complete coenzyme M biosynthesis pathway and does not contain a prophage or non-ribosomal peptide synthase genes. However, AbM4 has a large CRISPR region and several type I and type II restriction-modification system components. Unusually, DNA-directed RNA polymerase B′ and B′′ subunits of AbM4 are joined, a feature only previously observed in some thermophilic archaea. AbM4 has a much reduced complement of genes encoding adhesin-like proteins which suggests it occupies a ruminal niche different from that of M1.

## Introduction

Methane formed in the ruminant fore-stomach (reticulo-rumen) is a significant source of greenhouse gas emissions for countries that are reliant on ruminant-based agriculture. Methane is an end product of plant digestion in the reticulo-rumen, and is formed by methanogens belonging to the *Euryarchaeota* subgroup of the *Archaea*. Molecular surveys of ruminants have shown that small subunit ribosomal RNA gene sequences affiliated with species of the genus *Methanobrevibacter* predominate in most rumen microbiomes, on average making up almost two thirds of the rumen archaea [1]. Sequences are mainly associated with *M. gottschalkii* (33.6%) and *M. ruminantium* (27.3%), but also with *M. wolinii* (0.1%), *M. smithii* (0.1%) and other *Methanobrevibacter* spp. (0.5%). Development of mitigation strategies to reduce methane emissions from farmed animals is currently the subject of both scientific and environmental interest. Methanogens are producers of ruminant methane, therefore methane abatement strategies can either target the methanogens themselves or target the other members of the rumen microbial community that produce substrates necessary for methanogenesis. Genome sequencing has improved our knowledge of the processes that methanogens contribute to rumen function and is already providing information directly applicable to methane mitigation strategies based on vaccine and small-molecule inhibitor approaches [2,3]. Mitigation technologies for methane emissions from ruminants should target features that are conserved across all rumen methanogens, and be specific for methanogens so that the remaining rumen microbes can continue their normal digestive functions. Targeting ruminal methanogens using vaccine and small molecule inhibitor approaches needs to take into account the phylogenetic diversity covering the different groups of methanogens within the rumen and capture the inter-species diversity within a genus. Our group is sequencing the genomes of cultured representatives of rumen methanogens [4] to define their conserved features as targets and to understand their role in the ruminant environment for the purpose of developing methane mitigation technologies. Here, we report the complete genome sequence of *Methanobrevibacter sp.* AbM4.

## Classification and features

**Figure 1:**
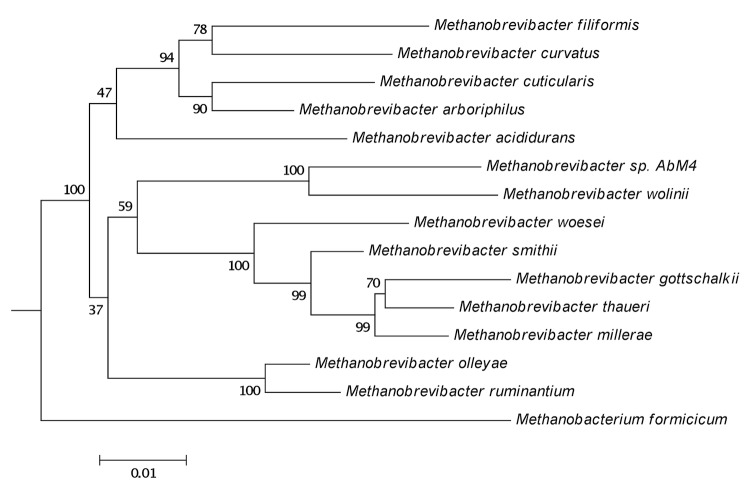
Phylogenetic tree showing the position of *Methanobrevibacter* strain AbM4 relative to type strains of other *Methanobrevibacter* species. The strains and their corresponding GenBank accession numbers for 16S rRNA genes are (type=^T^): *Methanobrevibacter gottschalkii* HO^T^, (U55238); *Methanobrevibacter thaueri* CW^T^, (U55236.1); *Methanobrevibacter millerae* ZA-10^T^, (AY196673); *Methanobrevibacter smithii* PS^T^, (U55233); *Methanobrevibacter woesei* GS^T^, (U55237); *Methanobrevibacter wolinii* SH^T^, (U55240); *Methanobrevibacter sp.* AbM4, (AJ550156); *Methanobrevibacter olleyae* KM1H5-1P^T^, (AY65201); *Methanobrevibacter ruminantium* M1^T^, (AY196666); *Methanobrevibacter curvatus* RFM-2^T^, (U62533); *Methanobrevibacter filiformis* RFM-3^T^, (U82322); *Methanobrevibacter cuticularis* RFM-1^T^, (U41095); *Methanobrevibacter arboriphilus* DH-1^T^, (AY19665); *Methanobrevibacter acididurans* ATM^T^, (AF242652) and *Methanobacterium formicicum* DSMZ1535^T^, (AF169245). The tree is based on these sequences aligned by the RDP aligner, and uses the Jukes-Cantor corrected distance model to construct a distance matrix based on alignment model positions without the use of alignment inserts, and uses a minimum comparable position of 200. The tree is built with RDP Tree Builder, which uses Weighbor [6] with an alphabet size of 4 and length size of 1,000. The building of the tree also involves a bootstrapping process repeated 100 times to generate a majority consensus tree [7]. *Methanobacterium formicicum* was used as the outgroup.

**Figure 2:**
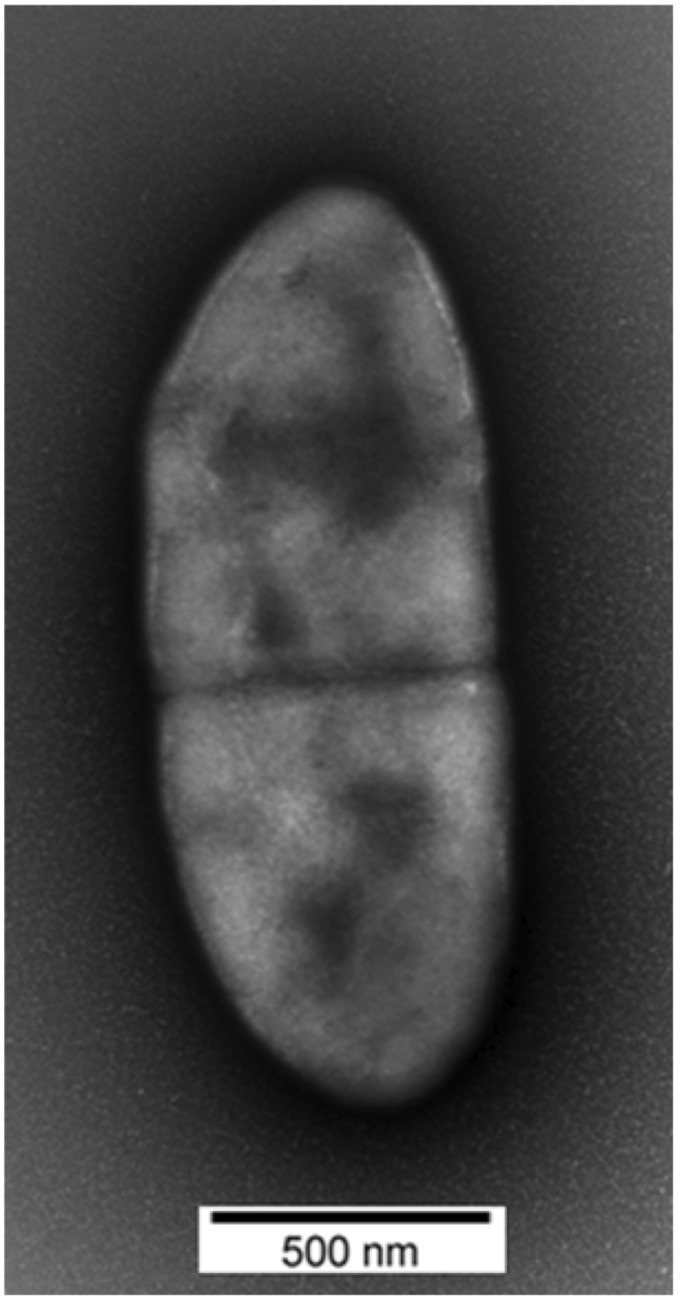
Transmission electron microscopy of negatively stained *Methanobrevibacter sp.* AbM4 cells.

*Methanobrevibacter sp.* AbM4 was isolated and purified from the abomasum of a sheep maintained as part of a study into effects of the nematode *Ostertagia circumcincta* on the abomasal environment [5] (Keith Joblin, personal communication). AbM4 is a member of the methanogenic archaea. It is a strict anaerobe and its hydrogenotrophic metabolism is characterized by its ability to produce methane from hydrogen, carbon dioxide and formate. A phylogenetic analysis of the AbM4 small subunit ribosomal RNA (ssrRNA) gene sequence places it closest to *Methanobrevibacter wolinii* and the sequence is approximately 95% similar to the *M. wolinii* type strain SH [Figure 1]. Although an ovine abomasal isolate, ssrRNA gene sequences identical, or with >97% similarity to that of AbM4 have also been reported among methanogen sequences derived from rumen contents of both sheep and cattle [8-10]. Searches of the Genbank and the Ribosomal Database Project databases also show sequences >97% similar to AbM4 occur in yak (Genbank accession JF807172), in sheep in Venezuela [11] and Western Australia [12], in alpacas [13] and Jersey dairy cows farmed in the USA [14], as well as in the feces of manatee in Florida, USA (Genbank accession HQ599703, HQ599742). The cellular morphology of AbM4 was determined by electron microscopy (Fig. 2). For this, AbM4 cells were grown on RM02 medium [2] and were negatively stained with 1% phosphotungstic acid, mounted on Formvar-coated copper grids. Grids were examined using a Philips model 201C electron microscope. AbM4 is a short rod and is not motile [Figure 2].

## Genome sequencing and annotation

### Genome project history

*Methanobrevibacter sp.* AbM4 was selected for genome sequencing on the basis of its phylogenetic position as a representative of organisms whose nearest relative is *M. wolinii*. AbM4 was isolated from a sample of sheep abomasal contents, whereas the type strain of *M. wolinii* SH was isolated from enrichment cultures of sheep feces [15]. AbM4 grows readily in broth cultures making it amenable to experimentation in the laboratory. A summary of the genome project information is shown in Tables 1 and 2.

**Table 1:** Classification and general features of *Methanobrevibacter sp.* AbM4

**MIGS ID**	**Property**	**Term**	**Evidence code**^a^
		Domain *Archaea*	TAS [16]
		Phylum *Euryarchaeota*	TAS [17]
		Class *Methanobacteria*	TAS [18,19]
	Current classification	Order *Methanobacteriales*	TAS [20-22]
		Family *Methanobacteriaceae*	TAS [23,24]
		Genus *Methanobrevibacter*	TAS [20,21]
		Species *Methanobrevibacter sp.* Strain AbM4	IDA
	Gram stain	Positive	IDA
	Cell shape	Short rod	IDA
	Motility	No	IDA
	Sporulation	No	IDA
	Temperature range	37-39 ^o^C	NAS
	Optimum temperature	38 ^o^C	NAS
	Carbon source	Acetate	IDA
	Energy source	H_2_ + CO_2_, formate	IDA
	Terminal electron receptor	CO_2_	IDA
MIGS-6	Habitat	Ovine abomasum, ovine and bovine rumen	IDA
MIGS-6.3	Salinity	Not reported	
MIGS-22	Oxygen	Strict anaerobe	IDA
MIGS-15	Biotic relationship	Symbiont of ruminants	NAS
MIGS-14	Pathogenicity	Not known as a pathogen	NAS
MIGS-4	Geographic location	Palmerston North, New Zealand	IDA
MIGS-5	Sample collection time	Not reported	
MIGS-4.1	Latitude	Latitude: -40.35 (40°21'00"S)	IDA
MIGS-4.2	Longitude	Longitude: +175.61 (175°36'36"E)	IDA
MIGS-4.3	Depth	Not reported	
MIGS-4.4	Altitude	30 m	

a) Evidence codes – TAS: Traceable Author Statement; IDA: Inferred from Direct Assay; NAS: Non-traceable Author Statement (i.e., not directly observed for the living, isolated sample, but based on a generally accepted property for the species, or anecdotal evidence) [25].

**Table 2:** Project information

**MIGS ID**	**Property**	**Term**
MIGS-31	Finishing quality	High-quality, closed genome
MIGS-28	Libraries used	3 kb mate paired-end library
MIGS-29	Sequencing platforms	454 GS FLX, titanium chemistry, Macrogen
MIGS-31.2	Fold coverage	234×
MIGS-30	Assemblers	Newbler
MIGS-32	Gene calling method	Glimmer and BLASTX
	Genome Database release	On publication
	Genbank ID	CP004050
	Genbank Date of Release	On publication
	BioProject ID	PRJNA157813
	Project relevance	Ruminant methane emissions

### Growth conditions and DNA isolation

AbM4 was grown in BY medium [26] with added SL10 Trace Elements solution (1 ml added l^-1^ ) [27], selenite/tungstate solution (final conc. of selenite and tungstate are 3 and 4 μg l^-1^ respectively) [28]; and Vitamin 10 solution (0.1 ml added to 10 ml culture before inoculation) [2]. H_2_ was supplied as the energy source by pumping the culture vessels to 180 kPa over pressure with an 80:20 mixture of H_2_:CO_2_. Genomic DNA was extracted from freshly grown cells using a modified version of a liquid N_2_ and grinding method [29]. Briefly, AbM4 cultures were harvested by centrifugation at 20,000 × *g* for 20 min at 4 ^o^C and cell pellets combined into 40 ml Oakridge centrifuge tubes and frozen at -80 ^o^C. The frozen cell pellets were placed in a sterile, pre-cooled (-85 ^o^C) mortar and ground to a powder with periodic addition of liquid N_2_. Buffer B1 (5 ml Qiagen Genomic-Tip 500 Maxi kit, Qiagen, Hilden, Germany) containing RNase (2 μg ml^-1^ final concentration) was added to the powdered cell pellet to create a slurry which was then removed. An additional 6 ml of B1 buffer was used to rinse the remaining material from the mortar and pestle and combined with the cell slurry, which was then treated following the Qiagen Genomic-Tip 500/G Maxi kit instructions. Finally, the genomic DNA was precipitated by addition of 0.7 vol isopropanol, and collected by centrifugation at 12,000 × *g* for 10 min at room temperature and re-dissolved in TE buffer (10 mM Tris-HCl, 1 mM EDTA pH 7.5).

### Genome sequencing and assembly

**Figure 3:**
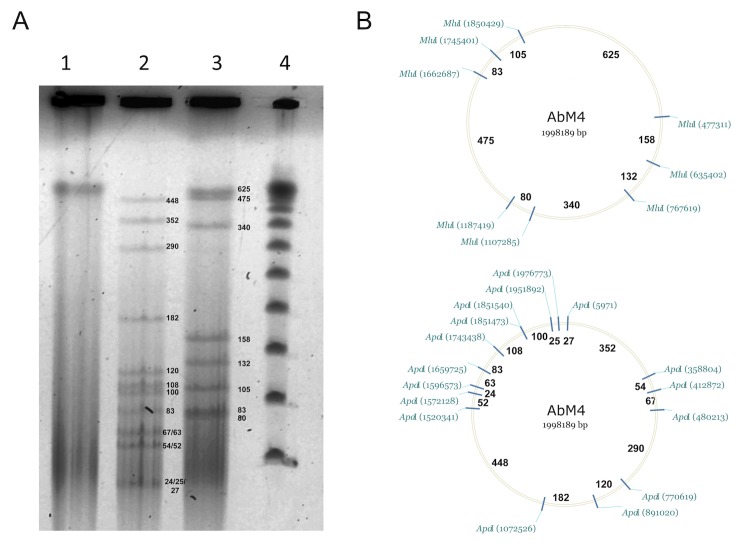
Pulsed field gel electrophoresis of *Methanobrevibacter sp.* AbM4 genomic DNA digested with restriction endonucleases (A) and *in silico* restriction enzyme maps (B). Panel A: Lane 1, undigested genomic DNA; Lane 2, digested with *Apa*I; Lane 3, digested with *Mlu*I; Lane 4, concatenated lambda marker (fragment sizes, from bottom in Kb, are 48.5, 97, 145.5, 194, 242.5, 291, 339.5, 388 and 436.5). Panel B: Maps of *Apa*I and *Mlu*I restriction endonuclease cleavage sites within the AbM4 genome.

The complete genome sequence of AbM4 was determined using pyrosequencing of 3kb mate paired-end sequence libraries using a 454 GS FLX platform with titanium chemistry (Macrogen, Korea). Pyrosequencing reads provided 234× coverage of the genome and were assembled using the Newbler assembler version 2.7 (Roche 454 Life Sciences, USA). The Newbler assembly resulted in 30 contigs across 4 scaffolds. Gap closure was managed using the Staden package [30] and gaps were closed using additional Sanger sequencing by standard and inverse PCR based techniques. A total of 80 additional reactions were necessary to close gaps, to improve the genome sequence quality and to ensure correct assembly and to resolve any remaining base-conflicts. Assembly validation was confirmed by pulsed-field gel electrophoresis (Figure 3) [2].

### Genome annotation

A GAMOLA/ARTEMIS [31,32] software suite was used to manage genome annotation. Protein-encoding open reading frames (ORFs) were identified using the ORF-prediction program Glimmer [33] and BLASTX [34,35]. A manual inspection was performed to verify or, if necessary, redefine the start and stop codons of each ORF. Assignment of protein function to ORFs was performed manually using results from the following sources; BLASTP [34] to both a non-redundant protein database provided by the National Centre for Biotechnology Information (NCBI) [36] and clusters of orthologous groups (COG) database [37]. HMMER [38] was used to identify protein motifs to both the PFAM [39] and TIGRFAM [40] libraries. TMHMM [41,42] was used to predict transmembrane sequences, and SignalP, version 4.1 [43] was used for the prediction of signal peptides. Ribosomal RNA genes were detected on the basis of BLASTN searches to a custom GAMOLA ribosomal database. Transfer RNA genes were identified using tRNAscan-SE [44]. Miscellaneous-coding RNAs were identified using the Rfam database [45] utilizing the INFERNAL software package [46]. The AbM4 genome sequence was prepared for NCBI submission using Sequin [47]. The adenine residue of the start codon of the Cdc6-1 replication initiation protein (Abm4_0001) gene was chosen as the first base for the AbM4 genome. The nucleotide sequence of the *Methanobrevibacter sp.* AbM4 chromosome has been deposited in Genbank under accession number CP004050.

## Genome properties

The genome of *Methanobrevibacter sp.* AbM4 consists of a single 1,998,189 bp circular chromosome with an average G+C content of 29%. A total of 1,730 genes were predicted, 1,671 of which were protein-coding genes. A putative function was assigned to 1,258 of the protein-coding genes, while the remaining protein coding genes were annotated as hypothetical proteins. The properties and statistics of the genome are summarized in Tables 3 and 4, and are compared with genomes of other sequenced gut methanogens for the order *Methanobacteriales* in Table 5.

**Table 3:** Genome Statistics

**Attribute**	**Value**	**% of total**
Genome size (bp)	1,998,189	100.00
DNA coding region (bp)	1,514,751	75.80
DNA G+C content (bp)	580,246	29.03
Number of replicons	1	
Total genes	1,730	100.00
RNA genes	45	2.60
rRNA operons	3	
tRNA genes	36	2.08
Protein-coding genes	1,671	96.58
Pseudogenes	14	0.80
Genes assigned to COGs	1,258	75.28
Genes with signal peptides	52	3.11
Genes with transmembrane helices	360	21.54

a) The total is based on either the size of the genome in base pairs or the total number of genes or protein-coding genes in the annotated genome.

**Table 4:** Assignment of AbM4 protein coding genes to COG functional categories

**Code**	**Value**	**%age**	**Descriptor**
J	144	8.62	Translation, ribosomal structure and biogenesis
A	0	0.00	RNA processing and modification
K	72	4.31	Transcription
L	84	5.03	Replication, recombination and repair
B	3	0.18	Chromatin structure and dynamics
D	6	0.36	Cell cycle control, cell division, chromosome partitioning
Y	-	-	Nuclear structure
V	23	1.38	Defense mechanisms
T	9	0.54	Signal transduction mechanisms
M	59	3.53	Cell wall/membrane/envelope biogenesis
N	4	0.24	Cell motility
Z	-	-	Cytoskeleton
W	-	-	Extracellular structures
U	11	0.66	Intracellular trafficking, secretion, and vesicular transport
O	38	2.27	Posttranslational modification, protein turnover, chaperones
C	140	8.38	Energy production and conversion
G	38	2.27	Carbohydrate transport and metabolism
E	117	7.00	Amino acid transport and metabolism
F	46	2.75	Nucleotide transport and metabolism
H	82	4.91	Coenzyme transport and metabolism
I	17	1.02	Lipid transport and metabolism
P	58	3.47	Inorganic ion transport and metabolism
Q	9	0.54	Secondary metabolites biosynthesis, transport and catabolism
R	184	11.01	General function prediction only
S	114	6.82	Function unknown
Not defined	413	24.72	No COG category assigned

**Table 5:** Complete genomes of *Methanobacteriales* from mammalian gut environments

**Species**	**Isolation source**	**Substrates**	**Genome size**	**Accession #**	**CDS**	**%GC**	**Reference**
*Methanobrevibacter sp.* AbM4	Ovine abomasum	H_2_ + CO_2_, CHOOH	2.0	CP004050	1671	29	This report
*Methanobrevibacter sp.* JH1	Bovine rumen	H_2_ + CO_2_, CHOOH	~2.06	BAGX02000001-54	1,786	28	[48]
*Methanobrevibacter ruminantium* M1	Bovine rumen	H_2_ + CO_2_, CHOOH	2.93	NC_013790	2217	33	[2]
*Methanobrevibacter smithii* PS	Sewage digester	H_2_ + CO_2_, CHOOH	1.85	NC_009515	1795	31	[49]
*Methanosphaera stadtmanae* MCB-3	Human feces	H_2_ + CH_3_OH	1.77	NC_007681	1534	28	[50]

**Figure 4:**
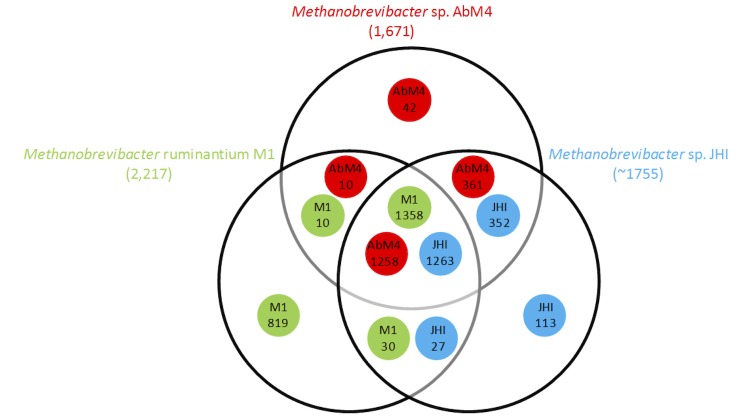
Venn diagram displaying gene conservation between the orfeome of *Methanobrevibacter ruminantium* M1, *Methanobrevibacter sp.* JHI, and *Methanobrevibacter sp.* AbM4. Analysis performed using GAMOLA [31] and OrthoMCL, Version 1.4 [51]. Numbers within circles refer to numbers of ORFs shared; numbers in brackets refer to total number of open reading frames predicted for a genome.

**Figure 5:**
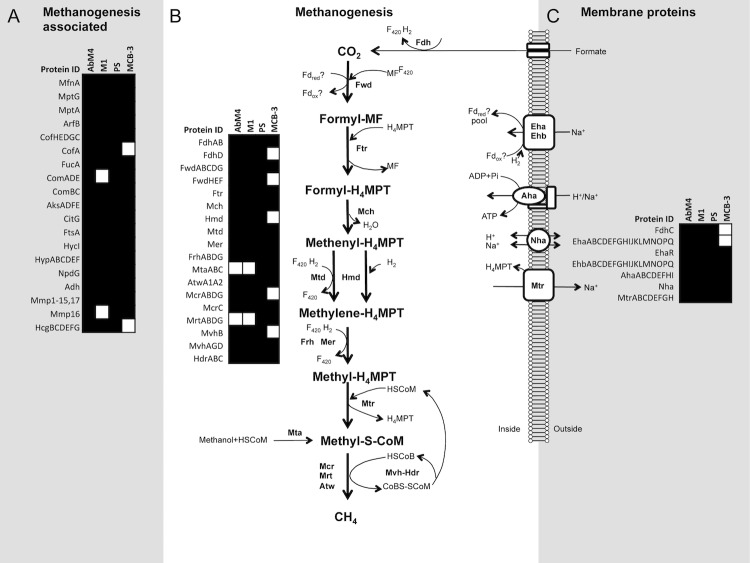
Comparison of predicted coding DNA sequence (CDS) content for methanogenesis associated functions from completed methanogen genomes found in mammalian gut environments. CDSs are arranged as methanogenesis-associated (A), methanogenesis (B) or membrane proteins (C). Genomes from *Methanobrevibacter sp.* AbM4, *M. ruminantium* M1 (NC_013790), *M. smithii* PS (NC_009515) and *Methanosphaera stadtmanae* MCB-3 (NC_007681) were used in the comparison. A white square indicates an absence, and a black square indicates a presence of the predicted protein listed at the left of each panel in the genome of the methanogen listed at the top of each panel.

Comparative analysis of the orfeomes of the rumen methanogen genome sequences, AbM4, *Methanobrevibacter ruminantium* M1 and the draft genome sequence of *Methanobrevibacter sp.* JHI [48], reveal that their gene content is largely comparable, particularly AbM4 and JHI (Figure 4). This suggests that the central metabolism and the methanogenesis pathway of these strains are similar. *Methanobrevibacter sp.* AbM4 is a hydrogenotrophic methanogen and the genes involved in the methanogenesis pathway, and associated functions are shown in Figure 5. The presence or absence of these genes is indicated within complete genomes of gut methanogens of the order *Methanobacteriales*. The methane formation pathway in AbM4 is very similar to that of M1 and each of the 7 enzymatic steps expected for the reduction of CO_2_ (or formate) through to methane using H_2_ is present. AbM4 and M1 are distinguished from the human gut methanogens, *Methanobrevibacter smithii* PS [49] and *Methanosphaera stadtmanae* MCB-3 [50], by the absence of the methanol:cobalamin methyltransferase genes (*mta*ABC) which mediate methanol utilization in these organisms. Hydrogenotrophic methanogens generally encode a methyl coenzyme reductase II (*mcr*II or *mrt*), an isoenzyme of the methyl CoM reductase I (*mcr*I) enzyme which is differentially regulated during growth [52] to mediate methane formation at high partial pressures of H_2_. Like M1, AbM4 contains only the McrI system for the final methyl-CoM reduction step in methanogenesis. PS contains both the McrI and II systems whereas MCB-3 contains only the McrII system. In the rumen, methanogens depend on fermentative microbes to supply H_2_, usually at very low concentrations, and AbM4 and M1 appear to have adapted their lifestyle for growth at low levels of H_2_ using the McrI system only. Comparable with M1, AbM4 contains several genes (two NADPH-dependent F_420_ dehydrogenase genes and three alcohol dehydrogenase genes) which may support growth on alcohols such as methanol and ethanol [2,53].

Genes unique to AbM4 as compared with other rumen methanogens (Figure. 4) and with an annotated function are enriched for type I and type II restriction-modification systems (Table 6). Compared to M1, AbM4 does not harbor a prophage but does contain a large (~16 kb) CRISPR region (sequence coordinates 134,105-150,352 bp). AbM4 has a greatly reduced complement of predicted adhesin-like proteins when compared to M1 (29 versus 105) and it lacks non-ribosomal peptide synthase genes that have been observed in M1. Although, AbM4 does not encode genes for biotin biosynthesis, it does contain a BioY transporter which would likely allow biotin uptake from the rumen environment. Unlike M1, AbM4 does not require exogenous coenzyme M for growth as it contains the full complement of CoM biosynthesis genes. A complete set of cobalamin biosynthesis genes are also present, although they are scattered throughout the genome, rather than being clustered together as in M1. Unusually, the DNA-directed RNA polymerase B′ and B′′ subunits of AbM4 are joined, a feature only previously observed in some thermophilic *Archaea* [54].

**Table 6:** Restriction-modification system genes unique to AbM4

**Locus tag**	**Annotation**
Abm4_0075	type I restriction-modification system M subunit HsdM1
Abm4_0077	type I restriction-modification system R subunit HsdR1
Abm4_0076	type I restriction-modification system S subunit HsdS1
Abm4_1018	type II DNA modification methylase
Abm4_0097	type II restriction endonuclease
Abm4_1017	type II restriction endonuclease
Abm4_0084	type III restriction protein restriction subunit

## Insights from the Genome Sequence

Overall, the genome of *Methanobrevibacter sp.* AbM4 is comparable to that of *M. ruminantium* M1 suggesting that the hydrogenotrophic, methane-forming metabolism of these rumen methanogens is highly analogous. The differences observed between AbM4 and M1 in the abundance of adhesin-like proteins indicates AbM4 invests less of its genetic resources on external interactions with its environment. The broader repertoire of cofactor and coenzyme biosynthetic genes of AbM4 also indicates that it is likely to be less dependent on other rumen microbes for the supply of cofactors for growth and survival in the rumen. These features suggest that AbM4 occupies a ruminal niche slightly different from that of M1. Although AbM4 does not constitute large methanogen populations, it is widely distributed in ruminant species under different rumen and gut conditions. The conserved nature of the AbM4 genes encoding these methanogenesis functions, as well as those encoding other potential targets for methane mitigation, indicates that AbM4 will be amenable to inhibition by small molecule inhibitors and vaccine-based methane mitigation technologies targeting these genes.
